# Surgical management of an unstable occipital condyle fracture in a dog using a patient-specific CNC-milled titanium implant: a case report

**DOI:** 10.3389/fvets.2026.1891073

**Published:** 2026-09-22

**Authors:** Narae Jeong, Hyunkyoung Kim, Cheongwoon Cho, Joohyun Jung

**Affiliations:** 1 Department of Veterinary Surgery, Ilsan Animal Medical Center, Goyang, Republic of Korea; 2 Department of Veterinary Surgery, College of Veterinary Medicine, Chungnam National University, Daejeon, Republic of Korea

**Keywords:** CAD, CNC milling, dog, occipital condyle fracture, occipitocervical stabilization, patient-specific implant

## Abstract

A 10-year-old castrated male Pomeranian was evaluated after a fall and presented with tetraparesis, dysphagia, and headturn. Magnetic resonance imaging (MRI) revealed a right occipital condyle fracture that caused brainstem compression and cerebellar infarction from a displaced bony fragment. According to the Anderson and Montesano classification in human medicine, this case was presumed to be Type 3, indicating instability, and surgical correction was considered. A bone fragment located in the medulla oblongata was left *in situ* owing to the potential risk of severe complications from removal. Occipitocervical stabilization surgery was performed to prevent further damage from instability. A patient-specific titanium implant was designed by CAD and fabricated by CNC milling, and screws were applied to the occiput and C1 vertebra using standard techniques, whereas a screw was inserted transversely into the C2 spinous process to minimize the risk of implant failure from neck flexion. Postoperative computed tomography (CT) confirmed proper implant placement without neural injury. Over time, the patient showed improvements in hypoglossal dysfunction and gait, and was discharged on postoperative day five. Occipital condyle fractures are rare in both veterinary and human medicine, with most cases classified as type 1 or 2, and typically do not require surgical intervention. To the best of our knowledge, this is the first peer-reviewed description of surgical stabilization of an occipital condyle fracture in a dog using a patient-specific, CNC-milled titanium implant. Conservative management of occipital condyle fracture and surgical stabilization of craniocervical junction fractures have been described previously, and the contribution of the present report lies in the implant design and fabrication workflow rather than in the surgical management of craniocervical injury *per se*.

## 1 Introduction

Occipital condyle fractures (OCFs) are uncommon injuries of the craniocervical junction, accounting for approximately 1–3% of blunt craniovertebral trauma in human patients (1–3). These fractures typically result from high-energy mechanisms, such as motor vehicle accidents or falls, and may be associated with significant instability or neurological deficits owing to the proximity of the brainstem and lower cranial nerves.

The Anderson–Montesano classification system is widely used to categorize OCFs into three types: type I (comminuted impaction fractures), type II (basilar skull fractures extending into the occipital condyle), and type III (avulsion fractures caused by alar ligament injury) (4). Type I and II fractures are generally considered stable and commonly managed conservatively, whereas type III fractures are potentially unstable owing to ligamentous disruption and may require surgical stabilization, particularly when associated with craniocervical misalignment or neural compression (4, 5).

In human medicine, most OCFs are treated non-surgically; however, surgical intervention is indicated in selected cases of instability or progressive neurological deficits (6, 7). Various surgical techniques have been described, including occipitocervical fusion, using plates, rods, and screw-based constructs (8). Despite these advances, optimal treatment strategies remain controversial, with some studies suggesting that radiographic evidence of instability may be more relevant than fracture classification alone in guiding management (9).

In veterinary medicine, OCFs have rarely been reported, and standardized diagnostic and therapeutic guidelines are lacking (10). The anatomical complexity of the craniocervical junction and proximity of critical neurovascular structures further limit surgical intervention, particularly in small-breed dogs (11). Furthermore, the scarcity of reported cases makes it difficult to establish evidence-based treatment recommendations.

Recent advances in computer-aided design and computer-aided manufacturing (CAD/CAM) have enabled the development of patient-specific implants, which may offer improved anatomical conformity and stability in complex regions such as the craniocervical junction (12–14). Such implants may be produced either additively, by techniques such as selective laser melting or electron beam melting, or subtractively, by computer numerical control (CNC) milling from wrought stock.

These two manufacturing routes differ in their resulting microstructure, fatigue behavior, and required post-processing, and are therefore not interchangeable; the implant used in the present case was produced by the subtractive route.

This study aimed to describe the surgical management of a type III occipital condyle fracture in a small-breed dog using a patient-specific CNC-milled titanium implant and to highlight the clinical decision-making, surgical technique, and outcome in the absence of established veterinary guidelines.

## 2 Case Report

### 2.1 Signalment and Clinical Presentation

A 10-year-old castrated male Pomeranian weighing 2.9 kg presented with an acute onset of severe neurological dysfunction, following a fall. The dog fell from approximately knee height while being held by the owner. It was presented to the referring veterinarian immediately after the fall and to our institution two days later.

Neurological examination revealed non-ambulatory tetraparesis with generalized hypertonia, severely delayed postural reactions in all four limbs, and increased bilateral spinal reflexes consistent with upper motor neuron lesions. Cranial nerve evaluation revealed a right-sided head turn and dysphagia, suggestive of lower cranial nerve involvement.

Computed tomography (CT) (Aquilion Lightning 80, Canon Medical Systems Corporation, Otawara, Tochigi, Japan) of the craniocervical junction revealed a right occipital condyle fracture with craniomedial displacement toward the foramen magnum, consistent with an Anderson–Montesano type III injury (4). Magnetic resonance imaging (MRI) (Achieva dStream 1.5T SmartSpeed Precise AI, Philips Healthcare, Best, the Netherlands) demonstrated compression of the medulla oblongata by the displaced fracture fragment along with a focal cerebellar lesion adjacent to the fracture site, suggestive of an infarction.

Given the instability associated with type III occipital condylar fractures and the presence of brainstem compression, surgical stabilization was performed. The displaced fracture fragment was left *in situ* because of the high risk of iatrogenic injury to adjacent neural and vascular structures.

### 2.2 Imaging

The preoperative imaging findings are summarized in Figures 1–3. CT of the craniocervical junction revealed a fracture of the right occipital condyle with craniomedial displacement of the bony fragment toward the foramen magnum (Figure 1, red arrow). MRI demonstrated compression of the medulla oblongata by a displaced fracture fragment (Figure 2, arrow). An adjacent focal cerebellar lesion showed T2 and FLAIR hyperintensity with mild contrast enhancement; diffusion-weighted imaging revealed high signal with corresponding low ADC values, findings most consistent with acute infarction (Figure 2, open arrow). 3D reconstruction of the craniocervical junction was used for surgical planning, including the generation of a patient-specific anatomical model and computer-assisted planning of implant positioning and screw trajectories (Figures 3A, B). An additively manufactured resin model of the patient's skull and cranial cervical vertebrae was produced for surgical rehearsal, with the CNC-milled titanium plate positioned on it during preoperative simulation (Figure 3C). Postoperative CT confirmed appropriate implant positioning without a cortical breach or vertebral canal intrusion (Figure 3D). At the 1-year follow-up, lateral and ventrodorsal radiographs (Figures 3E, F) confirmed that the implant and screws had remained stable throughout the follow-up period, with no radiographic evidence of screw loosening, backing-out, breakage, or implant displacement/migration.

**Figure 1:**
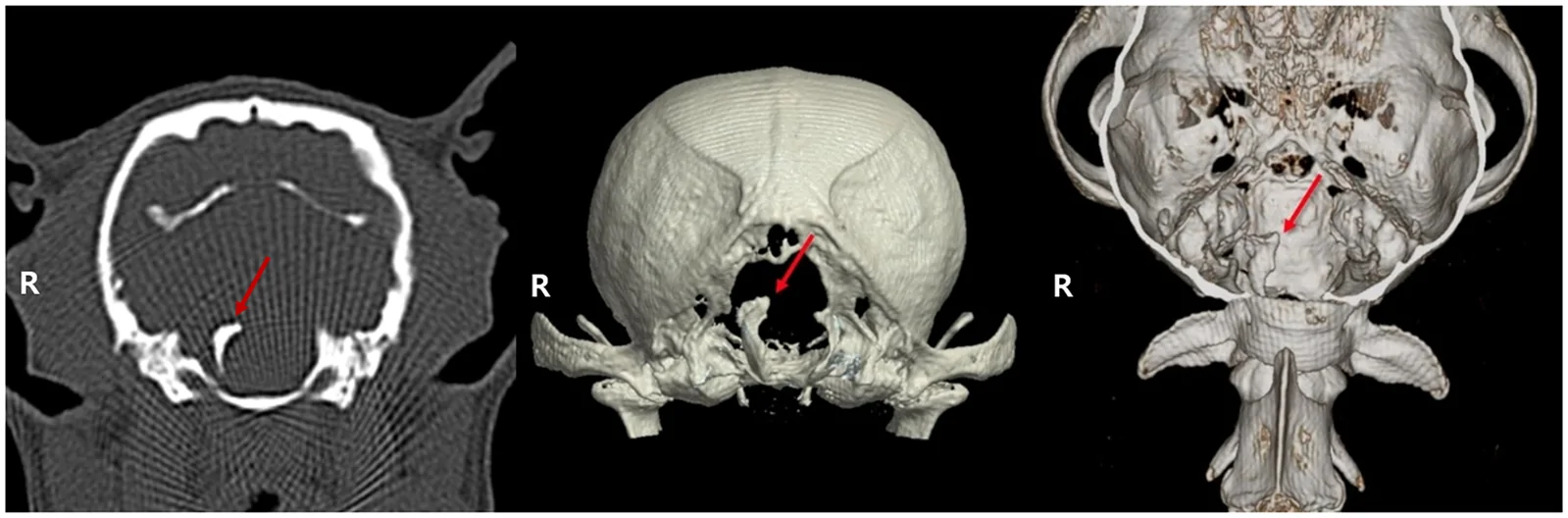
Preoperative computed tomography of the craniocervical junction demonstrating a right occipital condyle fracture with craniomedial displacement toward the foramen magnum. The red arrow indicates the displaced bone fragment originating from the occipital bone.

**Figure 2:**
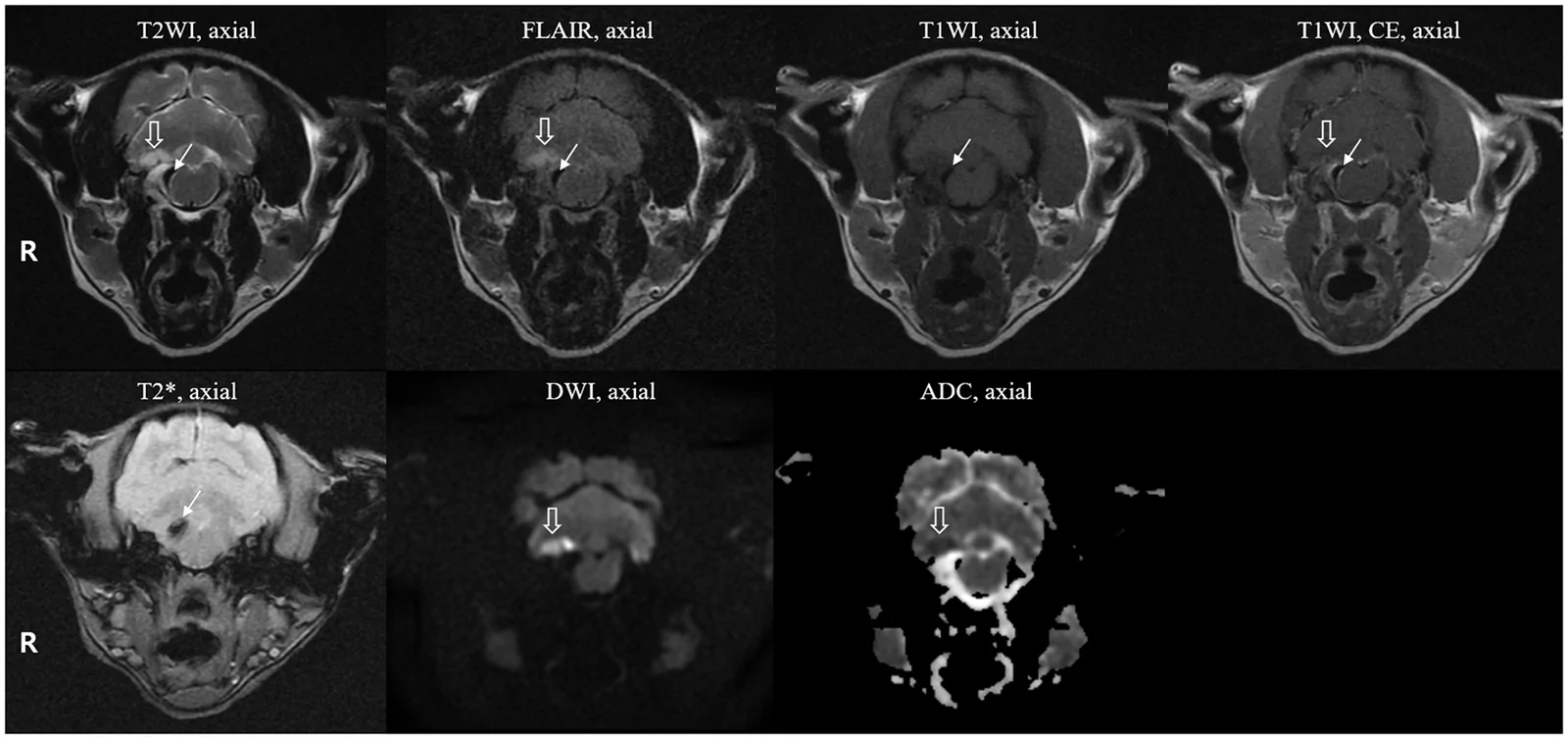
Magnetic resonance imaging demonstrating compression of the medulla oblongata caused by the displaced fracture fragment (arrow) and a focal cerebellar lesion adjacent to the fracture site (open arrow).

**Figure 3:**
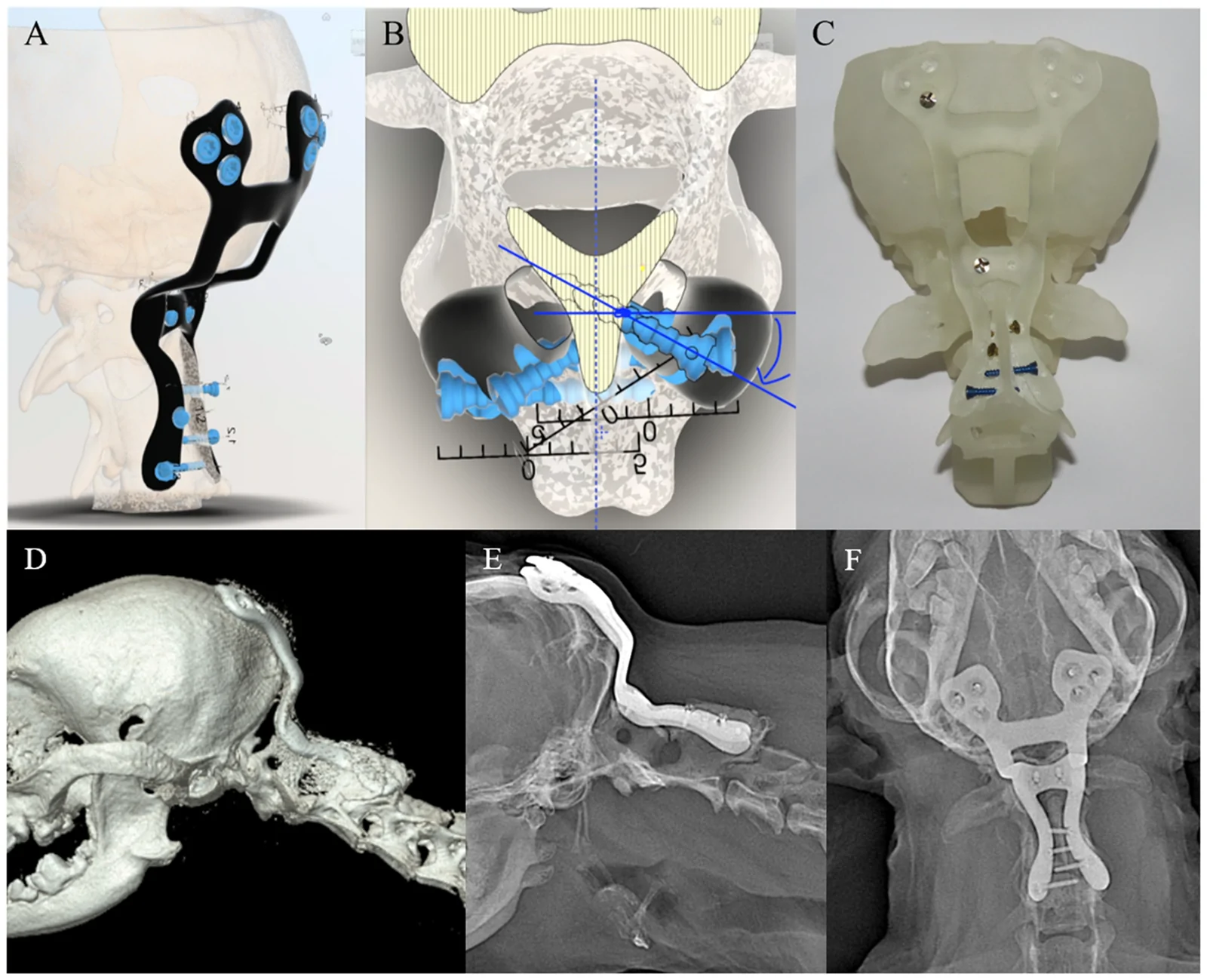
Preoperative planning and postoperative verification. **(A, B)** Patient-specific three-dimensional reconstruction of the craniocervical junction with computer-assisted planning of implant position and screw trajectories. **(C)** Additively manufactured resin model of the patient's skull and cranial cervical vertebrae, produced for surgical rehearsal, with the CNC-milled titanium plate positioned on it during preoperative simulation; the model was 3D-printed, whereas the plate was machined. **(D)** Postoperative computed tomography confirming that the implant and screws were positioned as planned, without cortical breach or vertebral canal intrusion. Lateral **(E)** and ventrodorsal **(F)** radiographs at 1 year confirmed a stable construct, with no implant or screw displacement.

### 2.3 Design and manufacturing of the custom titanium plate

Preoperative planning was performed using a CT-based 3D reconstruction. A patient-specific anatomical model was generated, and a customized titanium plate was designed to conform to the dorsal surfaces of the occiput, C1, and C2.

Additive manufacturing (3D printing) was used solely to produce a resin rehearsal model of the patient's skull and cranial cervical vertebrae for surgical simulation; it was not used at any stage in the fabrication of the titanium implant. The patient-specific plate and the accompanying rehearsal bone model were fabricated as a routine fee-based commercial service at a combined cost of KRW 800,000 (approximately USD 570), inclusive of delivery, with a turnaround of four days from design finalization to delivery. This cost is reported to assist readers in assessing the practical feasibility of the technique. The implant itself was produced by a subtractive process, and therefore parameters specific to additive fabrication—printing method (SLM/DMLS/EBM), powder specification, support structures, and hot isostatic pressing—are not applicable to it.

The implant was fabricated by subtractive machining. The design and manufacturing workflow is summarized in Table 1.

**Table 1:** Workflow for the design and manufacturing of the patient-specific implant fabricated by subtractive machining.

Stage	Software/ Equipment	Detail
Segmentation	3D Slicer (3D Slicer Foundation, United States)	Bone surface model; STL export
Plate design	3ds Max (Autodesk, United States)	Three-dimensionally contoured plate; STL export
CAM	hyperDENT (FOLLOW-ME! Technology GmbH, Munich, Germany)	Machining data preparation
Manufacturing	PT 1 (MANIX, Republic of Korea); 4-axis dental/metal milling machine	Ti-6Al-4V ELI (Grade 23)

Material and grade: Ti-6Al-4V ELI (Grade 23). No post-processing of any kind—surface finishing, heat treatment, annealing, or hot isostatic pressing—was performed after milling. Sterilization was by low-temperature hydrogen peroxide gas plasma. Fabrication of the plate together with the rehearsal bone model required four days from design finalization to delivery.

### 2.4 Surgical Procedure

Occipitocervical stabilization was performed 10 days after the injury under general anesthesia. Premedication consisted of midazolam (0.2 mg/kg IV). Anesthesia was induced with propofol (6 mg/kg IV to effect), and the trachea was intubated with a 4.0 mm armored endotracheal tube. Anesthesia was maintained with isoflurane (1.6%) in oxygen. Intraoperative analgesia was provided by a fentanyl constant-rate infusion, supplemented by local infiltration of bupivacaine (0.5 mg/kg) at the surgical site. Cefazolin (22 mg/kg IV) was administered perioperatively and repeated every 2 h intraoperatively. A balanced electrolyte solution (Plaju OP Inj.; JW Pharmaceutical, Seoul, Republic of Korea) was infused at 5 mL/kg/h. The dog was positioned in sternal recumbency with mild cervical flexion. The occipital bone, the atlas (C1), and the axis (C2) were exposed using a dorsal midline approach.

The plate was secured to the occiput using six 1.4 mm self-drilling screws, 4 mm in length. Fixation at C1 was achieved using two 1.2 mm cortical screws, 5 mm in length, placed in monocortical fashion. Fixation at C2 was achieved using four 1.2 mm cortical screws (5, 5, 8, and 10 mm, cranial to caudal), with the two cranial screws placed monocortically and the two caudal screws placed bicortically. A transverse screw orientation was applied at C2 to increase resistance to flexion and rotational forces (8). All screws were placed using a veterinary-specific fixation system (Arix Vet; Jeil Medical, Korea). The rationale for screw type and depth at each segment—including the use of self-drilling screws at the calvarium and the selection of monocortical placement where bicortical purchase would have interfered with the contralateral plate component—is detailed in Appendix.

Fabrication by 4-axis milling constrains the achievable geometry: an undercut, dorsally closed configuration at C2 cannot be machined in a single setup, and the plate was therefore designed with an open dorsal aspect at C2, its two arms descending on either side of the spinous process. A screw was placed transversely through the spinous process to link the two arms and to resist flexion and rotation, and polymethylmethacrylate was applied over the C2 component to bridge the open dorsal aspect and restore the flexural stiffness that a closed dorsal geometry would have provided. PMMA was thus used to compensate for a specific, manufacturing-imposed feature of the implant rather than as generic augmentation. The intraoperative findings are shown in Figure 4. The implant–bone interface at each segment, including screw diameter, length and cortical purchase, is detailed in Appendix.

**Figure 4:**
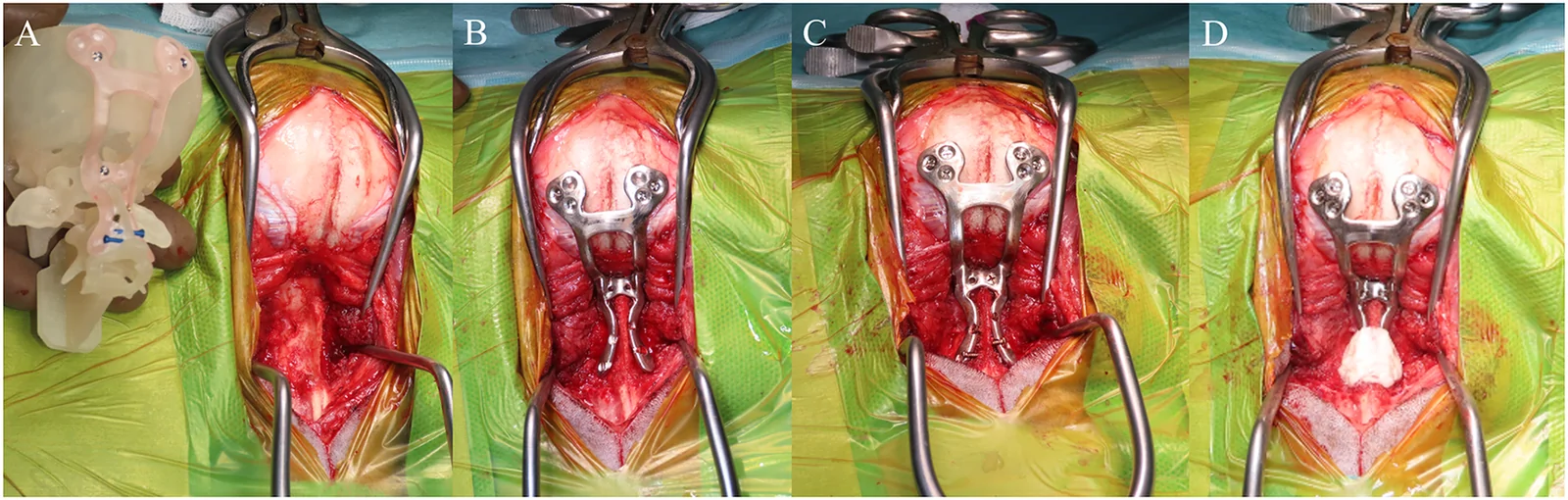
Intraoperative photographs demonstrating occipitocervical stabilization. **(A)** Surgical exposure of the occiput and cranial cervical vertebrae. **(B)** Placement of the patient-specific titanium plate prior to screw fixation. **(C)** Plate fixation to the occiput, atlas, and axis. **(D)** Application of polymethylmethacrylate to reinforce the construct.

Postoperatively, the dog received prednisolone (0.5 mg/kg PO q 12h) and gabapentin (10 mg/kg PO q 12h) for 10 days. A neck brace was applied, with strict exercise restriction for two weeks followed by partial exercise restriction for a further one month.

### 2.5 Recovery and Follow-up

The dog recovered uneventfully after anesthesia. Progressive neurological improvement was observed following surgery. At presentation, the dog was non-ambulatory tetraparetic, with hyperreflexia and hypertonia in all four limbs, a head turn, and dysphagia, consistent with a lesion localized to the caudal brainstem and cranial cervical spinal cord. At the 2-week postoperative re-examination, the dog was ambulatory with mild generalized ataxia as the only persisting gait abnormality, and voluntary prehension and swallowing had returned, with the dog eating unassisted. Dysphagia at presentation was characterized by marked oral spillage during prehension and mastication; however, the swallowing reflex was preserved and the dog was able to take food voluntarily. Enteral feeding tube placement was therefore not considered necessary, and nutrition was maintained by voluntary oral intake throughout hospitalization. Oral intake had normalized by the 2-week postoperative re-examination. Continuous recovery was noted over the subsequent months, with the dog becoming functionally ambulatory. Four months postoperatively, the dog exhibited a near-normal gait with only mild ataxia and was able to perform normal daily activities, including walking and running. At the 1-year follow-up, the patient maintained functional ambulation without deterioration. Serial radiographic follow-up through 1 year demonstrated maintenance of implant and screw position without evidence of implant migration or breakage. Radiography does not permit assessment of osseous fusion or of interval change in the retained fragment; follow-up CT was not obtained, and fusion status therefore remains undocumented. However, surgical stabilization resulted in a favorable neurological recovery and long-term functional outcomes.

## 3 Discussion

Occipital condyle fractures (OCFs) are rare injuries in veterinary medicine, and optimal management guidelines remain poorly defined due to the limited number of reported cases (10). Consequently, clinical decision-making is often extrapolated from human literature, where treatment strategies are primarily guided by fracture stability and neurological status. Under the Anderson–Montesano classification, Type III OCFs are considered potentially unstable due to alar ligament disruption, and surgical intervention is typically reserved for patients with radiographic instability, neural compression, or progressive deficits (4–6).

However, the Anderson–Montesano system was derived from human patients, in whom the craniocervical junction transmits axial load along a vertical weight-bearing axis. In quadrupeds, craniocervical loading is predominantly flexural rather than axial; thus, the biomechanical consequences of alar ligament avulsion may differ, and this classification has not been formally validated in canine patients. Therefore, the morphological correspondence of the present fracture to a Type III injury should be regarded as descriptive rather than implying equivalent instability. Nevertheless, in the absence of a standardized veterinary classification, applying this human-derived framework provided a useful descriptive rationale for assessing stability in this patient.

In the present case, preoperative imaging demonstrated displacement of the fracture fragment toward the foramen magnum, with associated compression of the medulla oblongata and neurological dysfunction. Although evaluation of the alar ligament typically benefits from specialized angled MRI sequences—which were not acquired preoperatively in this patient—alar ligament disruption was indirectly inferred from the fracture and displacement of the occipital condyle, which serves as its enthesis. Although direct fragment removal could be considered for decompression, this approach carries a substantial risk of iatrogenic injury given the confined anatomical constraints of the craniocervical junction (11). Based on these considerations, surgical stabilization without fragment removal was performed.

The favorable neurological recovery observed suggests that rigid internal stabilization alone may be sufficient to mitigate secondary mechanical stress and prevent further deterioration, even when a displaced fragment remains adjacent to the brainstem (9). These findings are clinically relevant as they support a less invasive surgical strategy in situations where direct decompression poses a significant risk.

However, postoperative cross-sectional imaging was limited to CT, which confirmed implant position but could not assess the neural parenchyma. Postoperative MRI was not performed, as an additional general anesthetic episode was not considered clinically justified in a recovering patient. Consequently, the retained fragment's ongoing relationship to the medulla oblongata and the evolution of the established cerebellar infarct could not be objectively documented. In the absence of postoperative neural imaging, the observed recovery cannot be causally attributed to stabilization alone; the resolution of vasogenic edema and maturation of the infarct over time represent equally plausible contributors. While stabilization reasonably prevented further fragment displacement and additional parenchymal injury, the relative contribution of each factor cannot be determined from this single case.

Another pivotal aspect of this case is the application of a patient-specific, CNC-milled titanium implant (12–14). The small size of the patient (2.9 kg) and the complex anatomy of the craniocervical junction severely limit the applicability of conventional fixation techniques. The customized implant allowed precise anatomical conformity, facilitating secure fixation across the occiput, C1, and C2. Furthermore, the incorporation of a transversely oriented screw at C2 provided increased resistance to flexion and rotational forces (8) while avoiding the technical risks associated with alternative screw trajectories.

Because the implant was machined from wrought Ti-6Al-4V ELI rather than built additively, its microstructure is homogeneous and its fatigue behavior more predictable than that of additively manufactured titanium, in which residual porosity and anisotropy arising from layer-wise consolidation are recognized concerns. The post-processing steps required to address these features of additive manufacture—stress-relief annealing and hot isostatic pressing—are correspondingly not applicable to a milled implant, and the absence of post-processing in the present workflow is therefore expected rather than a deficiency. The principal constraint of the subtractive route is geometric: undercut features cannot be machined in a single setup, which in the present case dictated an open dorsal configuration at C2 and the compensating use of a transverse screw and polymethylmethacrylate described in Section 2.4.

While OCFs managed conservatively without improvement and surgical stabilization of traumatic craniocervical junction fractures have been previously described, to the best of our knowledge, this report represents the first peer-reviewed description of surgical stabilization of an occipital condyle fracture in a dog using a patient-specific, CNC-milled titanium implant. The primary novel contribution lies in the patient-specific implant design and its fabrication workflow rather than in the management of craniocervical junction injury *per se*.

Nevertheless, the generalizability of our findings is limited by the fact that this report is based on a single case involving a 2.9-kg dog. Although this outcome suggests that stabilization-focused management is a viable option for select patients with OCFs, further studies are required to establish standardized veterinary diagnostic and treatment guidelines.

## Author's note

The patient-specific implant and rehearsal bone model were fabricated by Arix Vet as a routine, feebased commercial service. The manufacturer received no consideration beyond this standard fee, was not aware of this case report at the time of fabrication, and had no involvement in clinical decisionmaking, data interpretation, or manuscript preparation.

## Data Availability

The original contributions presented in the study are included in the article/supplementary material, further inquiries can be directed to the corresponding authors.

## Appendix

Occiput. Six 1.4-mm self-drilling screws were placed. Self-drilling rather than self-tapping screws were selected because self-tapping insertion carried a risk of uncontrolled penetration beyond the inner cortex in a calvarium of this thickness. Screw length was determined by preoperative simulation on the patient-specific model such that the screw tip achieved precise bicortical purchase of the occipital cortex without excessive intracranial protrusion. C1. Two 1.2-mm cortical screws, 5 mm in length, were placed monocortically, with length selected such that the tip abutted but did not penetrate the far cortex. C2. Four 1.2-mm cortical screws were placed, measuring 5, 5, 8, and 10 mm from cranial to caudal. The two cranial screws were placed monocortically, with the tip abutting the far cortex, because bicortical purchase at these positions would have caused interference with the contralateral plate component. The two caudal screws, where no such interference existed, were placed bicortically.
